# Functionalized Magnetic Nanoparticles for Alternating Magnetic Field- or Near Infrared Light-Induced Cancer Therapies

**DOI:** 10.3390/mi13081279

**Published:** 2022-08-08

**Authors:** Anilkumar Thaghalli Shivanna, Banendu Sunder Dash, Jyh-Ping Chen

**Affiliations:** 1 Department of Chemical and Materials Engineering, Chang Gung University, Kwei-San, Taoyuan 33302, Taiwan; 2 Department of Neurosurgery, Chang Gung Memorial Hospital at Linkou, Kwei-San, Taoyuan 33305, Taiwan; 3 Research Center for Food and Cosmetic Safety, College of Human Ecology, Chang Gung University of Science and Technology, Taoyuan 33305, Taiwan; 4 Department of Materials Engineering, Ming Chi University of Technology, Tai-Shan, New Taipei City 24301, Taiwan

**Keywords:** magnetic nanoparticles, magnetic hyperthermia, photothermal therapy, magnetic targeting, alternating magnetic field, near-infrared

## Abstract

The multi-faceted nature of functionalized magnetic nanoparticles (fMNPs) is well-suited for cancer therapy. These nanocomposites can also provide a multimodal platform for targeted cancer therapy due to their unique magnetic guidance characteristics. When induced by an alternating magnetic field (AMF), fMNPs can convert the magnetostatic energy to heat for magnetic hyperthermia (MHT), as well as for controlled drug release. Furthermore, with the ability to convert near-infrared (NIR) light energy to heat energy, fMNPs have attracted interest for photothermal therapy (PTT). Other than MHT and PTT, fMNPs also have a place in combination cancer therapies, such as chemo-MHT, chemo-PTT, and chemo-PTT–photodynamic therapy, among others, due to their versatile properties. Thus, this review presents multifunctional nanocomposites based on fMNPs for cancer therapies, induced by an AMF or NIR light. We will first discuss the different fMNPs induced with an AMF for cancer MHT and chemo-MHT. Secondly, we will discuss fMNPs irradiated with NIR lasers for cancer PTT and chemo-PTT. Finally, fMNPs used for dual-mode AMF + NIR-laser-induced magneto-photo-hyperthermia (MPHT) will be discussed.

## 1. Introduction

For many years, cancer has been ranked as one of the most challenging diseases to treat globally. The occurrence rate of cancer rose by 28 percent between 2006 and 2016, with worldwide cancer cases exceeding 17.2 million in 2016 and resulting in 8.9 million deaths [1,2]. Cancer can be defined as uncontrolled and irregular cell growth in any tissue in the human body. Today, the most widely used modalities for cancer treatment are surgery, chemotherapy, hyperthermia, immunotherapy, phototherapy, photodynamic therapy, and radiation therapy. [3]. Due to its ease and convenience, chemotherapy has been widely used clinically for cancer treatment, albeit with drawbacks and limitations [4]. For example, in chemotherapy, a toxic effect may arise due to its downside of non-specific targeting of cancer cells, which may be hazardous to normal or healthy cells [5]. Therefore, to overcome these limitations and achieve greater clinical efficacy for cancer treatment, a drug delivery system must be built for targeted delivery, or by combining chemotherapy with other types of cancer therapy. 

Nanostructured materials have been widely adopted in biomedical science to assist the creation of new methods of cancer therapy. They can resolve the disadvantages of traditional drug delivery methods and improve pharmacokinetics, thereby reducing side effects and improving performance. Due to the numerous advantages offered by functionalized nanomaterials, such as their high surface-to-volume ratios, passive tumor targeting via enhanced permeation and retention (EPR), versatility for surface modification with targeting ligands, and capability to load different drugs, they provide an alternative strategy in cancer treatment [6]. The essential nanomaterials for cancer therapies may include organic or inorganic nanoparticles, each with distinctive features [7]. To form organic nanoparticles, synthetic or natural polymers can be used. Many synthetic polymers have been studied in recent decades for nanomedicine applications, particularly in drug delivery. For such applications, these polymers must be non-toxic, biodegradable, and biocompatible. Two methods are being used to prepare drug-loaded polymeric nanoparticles, one is to encapsulate drugs within nanoparticles, where drugs are distributed throughout a polymeric matrix during formulation, and the other is to conjugate drugs to pre-formed nanoparticles [8]. The surfaces of polymeric nanoparticles can also be modified with targeting ligands or conjugated with polyethylene glycol to protect these particles form blood clearance [9]. The use of inorganic nanoparticles in medical applications is relatively recent, with their development occurring at the end of the last century. Inorganic nanoparticles can be used by functioning as photothermal agents or photosensitizers in cancer phototherapy [10,11,12]. Due to their unique physiochemical properties and excellent drug-loading capacity, inorganic nanoparticles stand out as ideal mediators in immunotherapy by serving as carriers to transport immunomodulatory agents for photoimmunotherapy [13].

The blood circulation time of nanoparticles is of great importance, since almost all nanoparticles will interact with the blood contents post-injection, irrespective of their final destination. Once they have entered the body, nanoparticles will be rapidly cleared from the bloodstream by interacting with the host’s immune system and will be engulfed by cells of the mononuclear phagocyte system (MPS) [14]. This natural defense system may result in a very short particle half-life in the blood (<1 min), and most of the injected dose will be cleared through the circulation within a few passes [15]. One solution to evade the MPS is to prevent nanoparticles from interacting with immune cells. This can be accomplished by coating the nanoparticles with a stealth shell or by allowing them to hide within red blood cells (RBCs). By coating them with a stealth shell of poly(ethylene glycol) (PEG), protein adsorption onto the nanoparticle surfaces may be reduced to prevent their recognition by immune cells and clearance from the blood [16]. However, the stealth coating tactics may impose restrictions on the functionality of the nanoparticles, when their functionality is built onto their surface [17]. The erythrocyte-assisted nanoparticle retention has been shown to efficiently prolong the circulation of nanoparticles recently, meaning the nanoparticles can be deposited onto the surfaces of RBCs or loaded inside RBCs to evade the MPS without controllable damage to the cell membrane [18,19]. In principle, this approach has the potential to prolong the nanoparticle circulation time in the blood from the long lifespan of the RBCs. However, it remains unclear whether the efficient retention of nanoparticles can be implemented without adversely affecting their functionality. A different solution without putting restrictions on the injected nanoparticles is to affect the MPS itself. For this purpose, the administration of toxic compounds that deplete macrophages can substantially prolong the nanoparticle circulation [20]. Alternatively, injecting large doses of organic or inorganic materials represents a less-radical approach to block the MPS function without macrophage elimination [21]. However, since the required dose required for administration is usually large, the dose-related toxicity may prevent its adoption in a clinical setting [22]. To solve these problems, a recent report involving the administration of a low dose of allogeneic anti-erythrocyte antibodies to temporally deplete erythrocytes in mice was shown to increase the circulation half-life of nanoparticles [23].

## 2. Thermal Cancer Therapy

**Figure 1:**
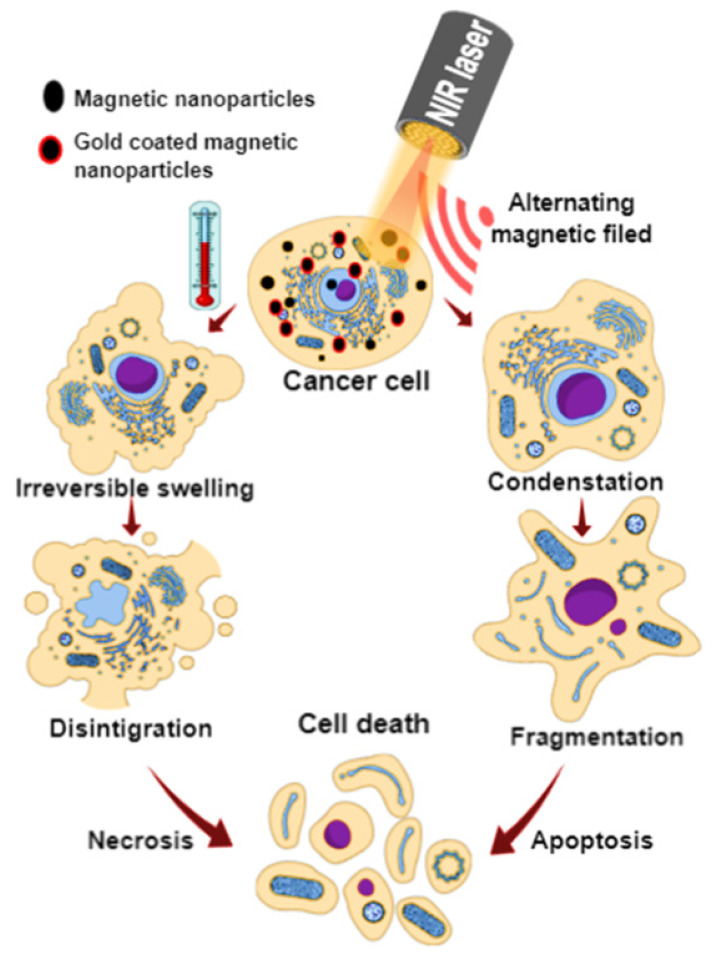
Magnetic nanoparticles or gold-coated magnetic nanoparticles induced by a near-infrared (NIR) laser or an alternating magnetic field (AMF) cause cancer cell death via apoptosis or necrosis by increasing the temperature.

Over the years, multiple devices representing different heat source types have been utilized in thermal cancer therapies, where temperature increases in vivo plays a significant role. By increasing the temperature at the tumor site above or equivalent to 43 °C for a specific period of time of up to 60 min, irreversible damage to tumor cells will occur. This hyperthermia-based cancer therapy can kill cancer cells via an apoptotic or necrotic pathway (Figure 1). The cell death is due to irreversible degeneration or the cessation of vital cell functions, leading to the collapse of the cellular integrity, while normal cells usually tolerate more at this temperature [24]. 

Throughout the clinical practice, hyperthermia can be employed alone or paired with other therapies such as chemotherapy, radiotherapy, and immunotherapy [3,25,26]. Hyperthermia can be employed in different ways, including local, regional, or whole-body hyperthermia. Local hyperthermia is commonly used to treat tumors in small body cavities, such as small tumors just beneath the skin. Regional hyperthermia is applied to broader regions than local hyperthermia, and is primarily used to treat whole organs or limbs. The usual form used for whole-body hyperthermia is the immersion of a body in a hot water bath or radiant heat or ultraviolet radiation, which is applied to metastatic tumors [26,27,28]. The temperature used in thermal ablation is higher than in hyperthermia, usually >50 °C, and the time duration is comparatively shorter, with treatment times usually being within 4 to 5 min [29]. Generally, for thermal ablation, 2 to 3 repeated cycles of treatments are performed to ablate the tumors [26,27]. For thermal cancer therapy, magnetic nanoparticles (MNPs) can fulfill a dual role due to their unique properties, such as in magnetic targeting under the guidance of an external magnetic field, acting as a thermal agent after induction with a high-frequency alternating magnetic field (AMF), or under near-infrared (NIR) laser irradiation. The AMF-induced tumor temperature increase leads to magnetic hyperthermia (MHT) in thermal cancer therapy. On the other hand, temperature increases in tumors induced by NIR lasers are used in cancer photothermal therapy (PTT). The main advantage of PTT when compared with conventional chemotherapy or radiotherapy is the ability to penetrate deep tissues with limited impact on the surrounding tissues, minimizing non-selective cell death [30]. Alternatively, the combination use of NIR lasers and AMFs leads to magnetic photothermia–hyperthermia or magneto-photo-hyperthermia (MPHT) for cancer magneto-photothermal therapy. In the following sections, we discuss the use of different functionalized magnetic nanoparticles (fMNPs) in MHT, PTT, and MPHT, as well as in combination cancer therapy incorporating thermal cancer therapy. 

## 3. Alternating Magnetic Field (AMF)-Induced Magnetic Hyperthermia (MHT)

The MNPs can be induced by an AMF at a specific frequency (f), amplitude (A), and magnetic field (H) based on the Néel–Brown relaxations mechanism to produce heat during MHT. Superparamagnetic nanoparticles are used for this purpose. The temperature increase rate is dependent on the nature of the biological medium and the specific absorption rate (SAR) of the MNPs [31]. The detailed heating mechanism has been described before [32]. The advantage of using MNPs for MHT is the possibility to generate heat in deep tumor tissues, and the applied AMF is not harmful to the human body, if H × f ≤ 5 × 10^8^ A m^−1^s^−1^ [33]. To meet the needs in the clinic, the application of MHT requires particle imaging technologies such as magnetic particle imaging [34]. For real-time monitoring and diagnosis, magnetic resonance imaging (MRI) can be performed with MNPs due to their high biocompatibility and excellent magnetic properties. Nonetheless, the main drawback of MHT is the requirement of a high dosage of MNPs (usually 1 to 2 M), which is much higher than those required for MRI. 

Nevertheless, AMF-induced MHT involves several challenges, including inconsistent results due to the aggregation of nanoparticles. Many studies have suggested that when placed in a biological medium emulating the cellular environment, the ability of MNPs to produce heat in response to AMF exposure is severely reduced [35,36]. This also happens when the heating capacity of the MNPs is largely reduced after internalization by cells, due to nanoparticle aggregation and dipolar interactions between nanoparticles [37,38]. Previously, MNPs were found to have a decreased SAR once internalized by cells, depending on the size, shape, and composition of the nanoparticles [39,40]. This diminished response of the MNPs to AMFs has been explained by the massive particle aggregation observed in vitro, which led to an unpredictable orientation in the lysosomes of cancer cells [41]. On the other hand, other studies have linked this effect to Brownian mobility restriction [42]. Indeed, most of the observed changes of the properties of MNPs due to particle aggregation could be explained by the restriction of the Brownian relaxation, as MNPs cannot respond to AMFs when the rotation of the MNPs is physically blocked [43]. Therefore, when the Brownian relaxation component is suppressed, the Néel relaxation component becomes the only possible heat induction mechanism [37]. Undoubtedly, the limitation associated with the aggregation of MNPs represents a main bottleneck in MHT, and a strategy must be developed to avoid nanoparticle aggregation for successful AMF-induced MHT in cancer treatment.

Therefore, the convergence of two or more modes of cancer therapy with MHT has been developed to overcome such limitations, for instance by combining chemotherapy with MHT for chemo-MHT to synergistically treat cancer [44]. By combining chemotherapy with MHT, a higher cancer cell death rate could be achieved than using fMNPs alone for thermally induced cell destruction. The release of chemotherapeutic drugs from fMNPs could also be externally regulated by an AMF by using thermal-sensitive functionalizing agents, after the magnetic targeted delivery of fMNPs to the tumors [45]. However, it should be noted that MHT is approved for human treatment only with iron oxide nanoparticles, and despite a successful prolonged overall survival rate being demonstrated in clinical trials, it has been a struggle to establish a clinical presence for this nanotechnology-based thermal cancer therapy [46].

### 3.1. Silica-Functionalized MNPs

Mesoporous silica (MS) is a porous material whose pore size lies within the mesoporous range of 2 to 50 nm. It has significant application in drug delivery owing to its large pore size. The MS can be derived from sources such as sodium silicate and alkoxide-like tetraethyl orthosilicate and is easily decomposed. The advantages of MS include its chemical and thermal stability, tunable pore size, as well as being readily available for surface modifications. The synthesis of MS involves simple processes such as the sol–gel method, and it can be surface-modified according to the required applications. Studies have shown that MS, in addition to being a high-capacity adsorbent, can hold drug molecules for a considerably long duration. Saavedra et al. investigated the MS=sphere-encapsulated MNPs (maghemite, γ-Fe_2_O_3_) for hyperthermia in vitro [47]. The synthesized magnetic MS matrix showed strong biocompatibility and reduced cell viability after induction with an AMF (100 kHz, 15,916 A m^−1^) for 45 min. In another study, Majeed et al. synthesized silica-coated MNPs and examined the anticancer efficacy of MHT using human cervical cancer cells [48]. After preparing Fe_3_O_4_ MNPs at 80 °C, which provided the highest specific absorption rate (SAR) at 111 Wg^−1^, SiO_2_ was coated on the surface to prepare core–shell Fe_3_O_4_–SiO_2_ MNPs to prevent the oxidation and agglomeration of the MNPs. Biocompatibility studies showed ~90% cell viability when tested with primary fibroblast cells L929 and HeLa cancer cells. As MHT is more pronounced for Fe_3_O_4_-SiO_2_ than for bare Fe_3_O_4_, the cytotoxicity studies showed a 27% higher cell death rate when cells were treated with Fe_3_O_4_-SiO_2_, under induction heating for 10 min with an AMF (26,659 A m^−1^, 250 kHz) when compared with uncoated Fe_3_O_4_. In another study, Kumar and co-workers also produced silica-coated multi-functional MNPs for real-time monitoring, diagnosis, and hyperthermia in cancer therapies [49]. In their study, manganese ferrite nanoassemblies (MNAs) were first synthesized using a polyol method. The MNAs were sequentially coated with two separate silica layers, a mixed silica layer containing a fluorescent dye rhodamine-B isothiocyanate (RITC) and a RITC-free silica layer in two sol–gel steps for the preparation of MNAs@Dye–SiO_2_@SiO_2_. The synthesized MNAs@Dye–SiO_2_@SiO_2_ showed superparamagnetic properties, enhanced colloidal stability, and good biocompatibility, with a size of around 100 nm. It also showed a lower magnetization density but increased SAR values for MHT. Upon treatment with MNAs@Dye-SiO_2_@SiO_2_ under an AMF (33.3 kA m^−1^), 80 to 85 percent approximate cell death rates were observed during in vitro cytotoxicity studies.

Renard et al. investigated hyperthermia cancer therapy in a solid tumor mice model using superparamagnetic iron oxide nanoparticles (SPIONs) trapped within silica microbeads [50]. Through their surface modification with silica, the SPIONs can prevent phagocytosis due to their repeated heating ability under magnetic induction. The synthesized SPIONs of silica microbeads (SSMB) were delivered through intratumoral (IT) injection into colorectal-tumor-bearing mice and exposed to an AMF (141 kHz) for 20 min, with 9 or 12 mT field strength. With experiments conducted under a 12 mT magnetic field strength, the tumor temperature could reach 47.8 °C with a 70% tumor necrosis rate based on the histology results. This could be compared with the 9 mT field strength, where the tumor temperature only reached 40 °C with no identifiable necrosis. By sacrificing the animals when the tumor size reached 10 times the initial tumor size, the median survival time could be increased to 37 days for mice treated with a 12 mT AMF, in contrast to 12 and 21 days for the control group without the IT delivery of SSMB, and for the treatment group with the IT delivery of SSMB but without the AMF exposure. Xu and colleagues produced magnetic-MS-modified and aminated (MMSNs-NH_2_) nanoparticles entrapping doxorubicin (DOX) and carboxyl-modified DNA_20_ (deoxyribonucleic acid with 20 bases) (DOX/MMSNs-NH_2_/DNA_20_) [51]. The synthesized formulations induced with an AMF (1.8 mT, 409 kHz) can produce heat from the superparamagnetic nature of MNPs and release drugs in a controlled manner. Confocal laser scanning microscopy confirmed the cellular internalization of fluorescein isothiocyanate (FITC)-labeled nanoparticles and DOX/MMSNs-NH_2_/DNA_20_ demonstrates synergistic cytotoxic effects against HeLa cells from in vitro studies. Lu et al. conducted a similar study with MNPs and DOX entrapped in hollow MS for controlled drug release and MHT [45]. In another study, Tian et al. entrapped Fe_3_O_4_ MNPs (size 15–20 nm) in MS nanoparticles (MMSN), which was then modified with a thermal-responsive copolymer poly-(NIPAM-co-MAA) and loaded with DOX [52]. The DOX-MMSN@P(NIPAM-co-MAA) demonstrated the good thermal induction properties under an AMF (1.2–1.8 mT, 409 kHz), which led to DOX release and enhanced the cell destruction ability towards HeLa cells due to its synergistic effect. In another study, Baeza et al. developed a model to study magnetically triggered drug release from a polymer-modified magnetic MS matrix [53]. A small molecule (fluorescein sodium salt) and a protein (soybean trypsin inhibitor, STI) were co-encapsulated in a nanocomposite loaded with maghemite (γ-Fe_2_O_3_) and surface-modified with a thermosensitive co-polymer poly-(ethyleneimine)-b-poly-(N-isopropylacrylamide) (PEI/NIPAM). Surface modifications with this polymer can aid in protein binding through electrostatic interaction or hydrogen bonding, and it can act as a temperature-sensitive gatekeeper for drugs. The formulation was investigated for its drug release kinetics induced by an AMF. The AMF can regulate drug release by opening and closing the pores on the surface of the silica matrix due to the phase change of the thermosensitive polymer. The same group also demonstrated a chemo-MHT model in C57BL/6 mice carrying EL4 murine lymphoma [54]. The MNPs were capped with oleic acid (OMNPs) and entrapped in an MS matrix (MS@OMNPs). A similar modification with a thermo-responsive polymer NIPAM was used to produce NIPAM@MS@OMNPs and to load DOX as a chemotherapeutic agent. The in vivo experiments demonstrated the synergistic effect of chemo and hyperthermia (thermal) therapy after exposing NIPAM@MS@OMNPs to an AMF at 105 kHz and 18 kA m^−1^. This synergy is due to the combination of enhanced DOX release and MHT. A review paper by Ansari et al. on MHT discussed a similar chemo-hyperthermia combination cancer therapy approach using MNP-loaded magnetic silica nanocomposites [55]. 

### 3.2. Polymer- and Dendrimer-Functionalized MNPs

Although many drugs have been developed for cancer treatment, their poor solubility in water, ineffective tumor targeting, and short half-life have limited their efficacy in destroying cancer cells. Polymers and dendrimers, as well as polymer nanocomposites, may play a significant role in this scenario, by serving as a drug carrier to meet these needs as an effective treatment modality. Xiao et al. studied magnetic nanorings modified with poly-ethylene-glycol methyl ether (mPEG) for MHT, and tested with subcutaneously implanted breast cancer cells in mice [56]. The nanocomposite possesses a ferromagnetic vortex domain with circumferential magnetization from the iron oxide magnetic nanoring (FVIO) structure, without stray fields. The FVIOs show excellent colloidal stability and minimum remanence and coercivity, as well as higher magnetic saturation than bare SPIONs. The FVIOs with about a 70 nm size (outer ring diameter) were modified with a biocompatible mPEG coating measuring 6–8 nm in thickness. After the IT administration of FVIO-mPEG (0.3 mg/cm^3^ dosage of MNPs) to tumor-bearing mice, the animal was exposed to an AMF (31,831 A m^−1^) for 10 min. The control group (administrated with saline) showed a 25-fold tumor size increase from its original size in four days. In contrast, the AMF-exposed group showed complete tumor elimination after six days, without tumor recurrence throughout the observation period up to 40 days. Another group carried out a similar study by developing PEGylated FVIOs and mediated with PD-L1 blockade as an immune checkpoint therapy for mild hyperthermia using a triple-negative breast cancer (TNBC) animal model in mice bearing 4T1 breast cancer cells [57]. Bae and his coworkers modified ferrimagnetic Fe_3_O_4_ nanocubes with chitosan oligosaccharide as a polymer shell, and used it as an efficient heat generator for cancer hyperthermia [58]. In another study, phosphorylated-mPEG was decorated on the surface of ferromagnetic Fe_0.6_Mn_0.4_O nanoflowers for T1 and T2 MRI contrast imaging and MHT antitumor thermotherapy in mice tumor models bearing MCF-7 cancer cells [59]. 

In clinical trials, hyperthermia treatments may face critical issues such as uncontrolled temperature increases, which must be monitored carefully for safety reasons. To properly regulate thermal energy via feedback from the local temperature changes in response to the external power supply, smart magnetic nanoparticles with intrinsically tunable heat generation capability were developed by Jing et al. [60]. The nanoparticles composed of Fe and Si were engineered to possess an adjustable magnetic transition temperature by tuning the exchange between Fe atoms via the incorporation of silicon atoms. The developed nanoparticles are biocompatible and endowed with controlled drug release ability under magnetic stimulus due to their combination with a thermosensitive polymer. Tang et al. used an intermediate timeset technique to monitor temperature increases with PLGA-modified iron oxide nanoparticles in MHT [61]. Both in vitro and in vivo studies with a 10-cm-diameter coil in an AMF (frequency: 513 kHz, output current: 28.2 A, output voltage: 361 V, output power: 8 kW) were performed. During controlled hyperthermia, the mice were exposed to the AMF for 40 s, after which the AMF was paused for 10 s and retreated for 10 s. Subsequently, the process was paused for 20 s, and the last two processes were repeated. The tumor temperature exceeded 55.5 ± 0.5 °C after 40 s and then fluctuated within a range of 64.5 ± 1.3 °C. In contrast, during continuous exposure to the AMF, the tumor temperature exceeded 90 °C within 2 min. These findings indicate that intermittent or controlled hyperthermia may prevent side effects such as skin burning or wound formation, but may still destroy the tumor at a desirable temperature under short exposure times. Ling et al. observed similar temperature regulation in the tumor with MHT, first with a bovine liver and later in xenograft mice carrying MB-231 breast cancer cells by injecting polymethylmethacrylate-Fe_3_O_4_ (PMMA-Fe_3_O_4_) [62]. Computed tomography images post-injection showed that PMMA-Fe_3_O_4_ was well retained in tumor tissues with total tumor eradication by injecting 0.1 mL 10% PMMA-Fe_3_O_4_ with an AMF exposure period of 180 s to reach a tumor temperature of 53.1 ± 3.2 °C. In a different study, Albarqi et al. produced hexagonal cobalt and manganese-doped MNPs clustered with polyethylene glycol-b-polycaprolactone (PEG-PCL) copolymers for intravenous (IV) delivery to mice bearing ES-2 ovarian tumors [63]. The biodistribution study confirmed the tumor targeting efficiency, with clustered MNPs entrapped in PEG-PCL accumulating in the tumor area 12 h after IV injection. The tumor temperature reached 42 °C after induction with an AMF (420 kHz, 26.9 kA m^−1^ and 30 min), as shown by a tumor heating efficiency analysis, suggesting that IV administration can result in the accumulation of the nanocomposites at the target site, and can lead to a sufficient temperature increase for tumor ablation. 

To target the tumor region via overexpressed folate receptors on the cancer cell surface, Koichiro et al. synthesized folic acid (FA) and PEG-modified SPIONs. They observed 1.3 μg Fe per gram of tissue mass 24 h after an IV injection, leading to temperatures of around 38 to 39 °C after AMF induction (H = 8 kA m^−1^ and f = 230 kHz) for 20 min [64]. Esmaeili and colleagues tested dendrimer-trapped SPIONs for localized hyperthermia and MRI diagnosis. The 3-aminopropyltriethoxysilane-modified MNPs were functionalized with polyamidoamine to form a dendrimer nanostructure. Various characterizations techniques verified that the synthesized nanostructure can produce MRI contrast images with good biocompatibility and an MHT-induced cytotoxic effect [65]. Salimi et al. carried out MHT with a polyamidoamine dendrimer (G4@IONPs) with MCF7 breast cancer cells and HDF1 human fibroblast cells. The cytotoxicity of the synthesized nanoparticles was low but the viability of the cancer cells after being incubated with G4@IONPs decreased significantly under MHT [66]. 

Chen et al. developed a nanocomposite using biocompatible polydopamine (PDA) to modify Fe_3_O_4_ core–shell nanoparticles (Fe_3_O_4_@PDA), further modified with a metal–organic structure of zeolitic imidazolate frameworks (ZIFs-90). In Fe_3_O_4_@PDA@ZIFs-90, the porous shell (ZIFs-90) acts as a drug carrier and the core–shell Fe_3_O_4_@PDA acts as a growth inhibitor to ZIFs-90 [67]. These Fe_3_O_4_@PDA@ZIFs-90 nanoparticles can be loaded with DOX (DOX@Fe_3_O_4_@PDA@ZIFs-90) with a very high payload and an average size of 200 nm. Furthermore, this multifunctional nanocarrier system shows pH-triggered DOX release and can reach hyperthermia conditions in an AMF (409 kHz, 18 mT), which demonstrates the higher anticancer efficiency than when using chemotherapy or MHT alone. The combination chemo-MHT treatment seems to have a synergistic effect, with enhanced cytotoxicity towards HeLa cells in vitro. The group led by Reyes used two polymer-coated magnetic nanorods (MNRs) as drug carriers, and proved that the nanocomposites can release drugs in response to the heat generated from AMF induction [68]. The MNRs, namely magnetite (Fe_3_O_4_) and maghemite (γ-Fe_2_O_3_), were synthesized using co-precipitation and hydrothermal methodologies, followed by modifications with poly(ethyleneimine) (PEI) and poly(sodium 4-styrenesulfonate) (PSS) with a layer-by-layer polymer coating and loading with an antitumor drug DOX. The MNRs with adjustable sizes of 15 to 45 nm (Fe_3_O_4_) or 64 to 530 nm (Fe_2_O_3_) were synthesized under different synthesis conditions. After induction with an AMF (100–200 kHz, 10–20 kA m^−1^), the polymer-modified MNRs showed a substantial hyperthermia effect and 50% DOX release within 4 h in acidic media. The same group also used other types of MNPs and biocompatible polymers for the delivery of another antitumor drug (gemcitabine) [69,70]. In another study, Rana et al. used in situ polymerization to crosslink a polyaniline shell on carboxyl PEG-modified Fe_3_O_4_ nanoparticles to load the anticancer drug DOX and studied the pH-dependent drug release [71]. These formulations demonstrated a high drug payload, excellent intracellular uptake, and sustained drug release, indicating that chemo-MHT can significantly lead to the death of tumor cells in vitro.

Both in vitro and in vivo studies with drug-loaded and polymer-modified MNPs were conducted by Qu et al. by using MNPs as triggering agents for drug release, as MHT agents, and as external magnetic-field-guided targeting agents [72]. The Mn-Zn ferrite MNPs (MZF-MNPs) were modified with a thermosensitive amphiphilic polymer polylactide-b-poly(N-isopropylacrylamide-co-N,N-dimethylacrylamide) (PLA-b-poly(N-co-D)). A magneto-thermosensitive nanocomposite CPT/MTRN, formed by co-encapsulating hydrophobic MNPs and a chemotherapeutic drug camptothecin (CPT) in the copolymer, demonstrated a strong thermal effect induced by an AMF (114 kHz and 89.9 KAm^−1^) for controlled drug release. The effective synchronism of the thermo-chemotherapy was achieved from the enhanced cytotoxicity due to the CPT release and MHT, as demonstrated from in vitro studies using SK-OV-3 ovarian carcinoma cells and HepG2 hepatocellular carcinoma cells. In a follow-up study, the same groups synthesized another thermosensitive random copolymer 6sPCL-b-P(MEO_2_MA-co-OEGMA) from the copolymerization of 2-(2-methoxyethoxy) ethyl methacrylate (MEO_2_MA) and oligo (ethylene glycol) (OEGMA). The critical phase transition temperature of the co-polymer could be regulated by varying the molar ratio of the two monomers during synthesis. The copolymer was combined with MZF-MNPs and DOX to synthesize MTRN/DOX via self-assembly for the thermo-chemotherapy of liver cancer [73]. The nanocomposite exhibited controlled drug release when induced with an AMF due to the magnetic heating effect, and the lower critical solution temperature of the copolymer was controlled at 43 °C, a temperature at which the tumor cells were sensitized to chemotherapy. From the in vitro studies, the MTRN/DOX showed good magnetothermal effects and could be magnetically guided to enhance the sensitivity of Huh-7 cancer cells to DOX. From animal studies, the IV administration of MTRN/DOX + magnetic targeting + AMF resulted in a substantial reduction in tumor size in Huh-7 tumor-bearing mice compared to other treatment groups. During hyperthermia treatment in vivo, mice in the MTRN/DOX + magnet group experienced the most pronounced temperature change (52 °C) within 20 min due to the magnetic targeting effect. In contrast, mice in the MTRN/DOX group showed a lower temperature increase (42 °C in 20 min). This was supported by the greater accumulation of fluorescence signals associated with MTRN/DOX in the tumor area, while less was found in vital organs when guided by an external magnet for magnetic targeting. In comparison, the other two groups (free DOX and MTRN/DOX) displayed less fluorescence intensity in the tumor region and more fluorescence intensity in the vital organs. Undoubtedly, this efficiency was provided by using a multifunctional nanocomposite in tandem with chemo-magnetic hyperthermia therapy, implying that this combination approach is a promising cancer therapy modality.

### 3.3. Targeting Agent and Liposome-Functionalized MNPs 

**Figure 2:**
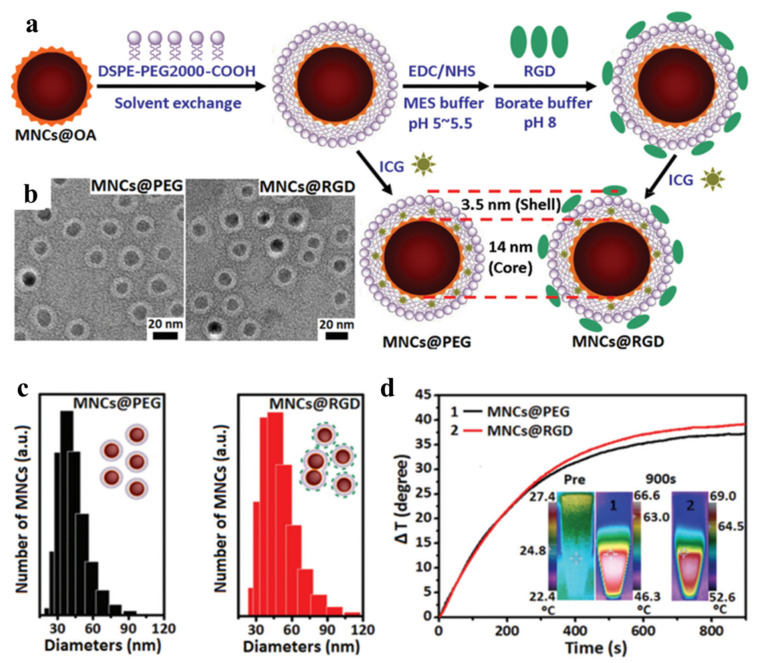
(**a**) The oleic acid (OA)-modified Mn-Zn ferrite nanocrystals (MNCs@OA) were coated with DSPE-PEG2000-COOH through hydrophobic interactions to form MNCs@PEG with entrapped ICG. The peptide ligand arginine–glycine–aspartic acid (RGD) was covalently linked to the nanocomposite to form MNCs@RGD. (**b**) The transmission electron microscope (TEM) images of core–shell structured MNCs after negative staining with 2% phosphotungstic acid reveal a white lipid shell layer covering a core of MNCs. (**c**) The size distribution histogram of MNCs measured from a dynamic light scattering image (DLS). (**d**) The in vitro temperature profile of MNCs (2 mg Fe/mL) after induction with an alternative magnetic field (AMF) at 390 kHz and 2.58 kA m^−1^. The insert shows the near-infrared (NIR) thermal camera images. Reprinted/adapted with permission from Ref. [76]. 2016, The Royal Society of Chemistry.

In general, targeting agents such as peptides, ligands, lipids, aptamers, and antibodies can be conjugated to nanoparticles, with the aim of binding them to overexpressed receptors on cancer cell surfaces. Hyaluronic acid (HA) is, for example, an ligand that can bind overexpressed CD44 receptors on U87MG brain tumor cells [74,75]. Jun et al. synthesized multimodal imaging nanocomposites for targeted hyperthermia using peptide-modified Mn-Zn ferrite nanocrystals (MNCs) [76]. Using arginine-glycine-aspartic acid (RGD) as a targeting ligand, the oleic-acid-coated MNCs were modified with phospholipid-PEG (DSPE-PEG2000-COOH) through hydrophobic interactions to synthesize MNCs@PEG, which could be further covalently linked with RGD to form MNCs@RGD (Figure 2). Indocyanine green (ICG) was trapped in the phospholipid-PEG layer for dual fluorescence imaging and MRI. From the in vivo studies, after induction with an AMF at a 390 kHz frequency, the tumor surface temperature could reach ~44 °C, which was sufficient to cause tumor cell apoptosis and prevent the angiogenesis of the tumor tissue. In another study, the core–shell gold-coated Zn-doped iron oxide MNPs were developed by Shah et al. and conjugated with a pro-apoptotic amphipathic tail-anchoring peptide (ATAP) for adjuvant targeted thermal–hyperthermic therapy [77]. With malignant brain and metastatic breast cancer cells, the multifunctional nanoparticle could target the tumor. With subsequent hyperthermia treatment resulting in mitochondrial dysfunction, an increased cancer cell apoptosis rate was achieved. Pala et al. used antihuman epidermal growth factor (HER2) aptamers to modify dextran-decorated ferric oxide MNPs (Ap-MNPs) and tested the targeting efficacy using AMF-induced hyperthermia [78]. Two separate cell lines were selected to assess the targeting efficiency of Ap-MNPs, whereby one overexpresses the HER2 receptors (human SK-BR3 cell lines) and the other does not (U87MG brain tumor cell lines). The in vitro experiments found that the Ap-MPNs showed higher cytotoxicity against HER2-overexpressing SK-BR3 while U87MG showed higher cell viability. A 90-fold lower dose of Ap-MNPs compared to bare MNPs showed a 50% hyperthermic killing effect on SK-BR3 cells, while U87MG was almost 100% viable. Using mAb-conjugated iron oxide MNPs, DeNardo et al. performed a specific tumor-targeting thermal therapy in xenograft mice bearing HBT3477 breast cancer cells [79]. Balivada et al. compared the IT and IV administration of ligand-modified MNPs in a B16-F10 mouse model [80]. In their study, core–shell Fe/Fe_3_O_4_ nanoparticles were modified with TCPP (4-tetracarboxyphenyl porphyrin) and conjugated with dopamine–oligoethylene glycol as a ligand. The in vivo studies in the mouse model show significant antitumor effects in a murine B16-F10 melanoma. The findings suggested that after repeated short AMF exposure, low-dose ligand-modified nanoparticles administered intravenously or intratumorally can significantly reduce the size of subcutaneously implanted B16-F10 tumors in mice.

Liposomes have a unique structure and can load cargos in their aqueous core surrounded by a lipid bilayer. The properties of liposomes can be modulated by selecting an appropriate lipid composition in the lipid bilayer. However, conventional liposomes used for the delivery of chemo-drugs have certain limitations, whereby the systemic delivery of high doses of drug-loaded liposomes lacking targeting ability towards tumor sites may result in drug resistance and other undesirable consequences [81]. By encapsulating MNPs in liposomes, the magnetic liposomes can be made to be thermosensitive by incorporating a temperature-sensitive lipid in the lipid bilayer. The encapsulated chemo-drugs in magnetic liposomes may offer controlled drug release after induction with an AMF. Several studies have been undertaken along this line. For example, Ferreira et al. created magnetic thermosensitive liposomes (MTLs) for hyperthermia-controlled drug release by loading iron oxide in the aqueous core and gemcitabine in the bilayer of the liposomes [82]. As a result, the MTLs released 70% of the gemcitabine within 5 min of mild hyperthermia caused by AMF, whereas at 37 °C only 17% of the gemcitabine was released after 72 h. Furthermore, magnetic liposomes (MLs) or MTLs can been further modified with targeting ligands and antibodies for dual targeting purposes, which can result in a synergistic targeting effect. Babincova et al. conducted a study in a rat C6 glioma by modifying the surface of MTLs with folic acid and by loading DOX into MTLs as an anticancer drug for chemo-MHT cancer therapy [83]. The magnetically targeted nanodrug showed a potent antitumor effect in vivo by reducing the tumor growth rate. The HA is a ligand that can bind to the CD44 receptor in U87MG brain tumor cells. This molecule was chosen by Jose et al. to modify liposomes with HA-grafted polyethylene glycol (HA-PEG) for in vitro studies [84] and by Anilkumar et al. for in vivo studies [74]. The findings from both studies supported the dual targeting ability of HA-modified MLs. In another study, Lu et al. prepared MTLs that were loaded with the anticancer drug Camptosar (CPT-11) and conjugated with an epidermal growth factor receptor (EGFR) antibody Cetuximab (CET) for dual targeted and AMF-induced drug delivery in a mouse intracranial tumor model [85]. As brain tumors overexpress EGFR, whereas normal tissues do not, a dual targeting strategy (magnetic + CET) was shown to increase the antitumor efficacy of the nanodrug from both in vitro and in vivo studies. A comprehensive review of the methods used to prepare MLs, as well as hyperthermia-based multi-modal cancer therapy, has been published [3].

### 3.4. Other Nanomaterial-Functionalized MNPs 

Several other nanomaterials for MHT were used to modify MNPs and tested for AMF inductions. Xu et al. developed a biodegradable and injectable Fe_3_O_4_-containing calcium phosphate cement (MCPC) for MHT tumor ablation [86]. After the IT administration of MCPC (0.36 g) in mice, the AMF induction (frequency: 626 kHz; output current: 28.6 A; coil diameter: 3 cm) for 180 s fully flattened the tumors by increasing the intratumoral temperature within 180 s. Another study by Cho and co-workers studied enhanced MHT by incorporating Fe_3_O_4_ nanocube (20 nm) into stabilized bovine serum albumin (BSA-NCs) [87]. The fMNPs showed a high SAR (109.8 ± 12.8 W g^−1^ at 512 kHz and 10 kA m^−1^) in U87MG human glioblastoma tumor cell-bearing mice. Treatment by MHT with an AMF for 3 consecutive days following the IV delivery of fMNPs (5 mg Fe/kg) led to retarded tumor progression. To enhance the technical application of MHT agents in clinical applications, the aggregation of MNPs could be reduced using surface-modifying MNPs with human-like collagen protein (HCP) to improve the SAR value [88]. Three uniform MNP particles with different sizes (8, 17, and 24 nm) were synthesized and modified with HCPs (HCP-MNPs). Compared to bare MNPs, the HCP-MNPs showed similar increases in AMF-induced temperature at 44,564 Am^−1^ and 360 kHz with excellent biocompatibility when tested with BHK-21 baby hamster kidney cells. This compatibility was also confirmed in vivo in mice through the subcutaneous injection of HCP-MNPs, whereby a minimum inflammatory response was noted in contrast to bare MNPs, which showed pronounced phagocyte activity. Kandasamy et al. conducted MHT for liver cancer treatment with fMNPs [89]. In their study, the MNPs were modified with different short-chain molecules, 1,4-diaminobenzene (1,4-DAB), 4-aminobenzoic acid (4-ABA), 3,4-diaminobenzoic acid (3,4-DABA), and mixtures of terephthalic acid (TA)/pyromellitic acid (PMA)/trimesic acid (TMA)/2-aminoterephthalic acid (ATA). All formulations had strong magnetic properties and water-dispersive ability, and only 4-ABA, 3,4-DABA, 1,4-DAB, and 4-ABA-TA-coated MNPs showed increased saturation magnetization values (Ms = 55–71 emu g^−1^). For fMNPs modified by 3,4-DABA, a rapid temperature increase was observed at a lower concentration (0.5 mg/mL) following their induction with an AMF, which led to 61–88% cytotoxicity for the HepG2 liver cancer cells. Kim et al. compared the characteristics of starch- and chitosan-modified MNPs. They found that chitosan-modified fMNPs had a higher saturation magnetization value (Ms = 25.6 emu g^−1^) compared to starch-modified fMNPs (Ms = 16.4 emu/g), which also showed higher temperature changes under AMF exposure (ΔT = 23 °C vs. 12 °C) [90]. Additionally, from the study of the biocompatibility with L929 cells, it was revealed that chitosan-modified fMNPs showed higher cell viability compared to starch functionalization. To combine the chemotherapeutic effects to magnetic hyperthermia using biocompatible scaffolds, a study from Pellegrino’s group loaded iron oxide nanocubes and DOX within the polycaprolactone nanofibers, which could be activated under an AMF [91]. The heat caused by MHT and DOX release when the scaffold was exposed to MHT treatment synergistically reduced the cancer cell viability as compared to drug release only and MHT without loaded drugs. The studies using functionalized MNPs for AMF-induced MHT and chemo-MHT cancer therapy are summarized in Table 1.

## 4. Near Infrared (NIR)-Light Induced Photothermal Therapy (PTT)

In photothermal therapy (PTT) for cancer, organic and inorganic photothermal agents (PAs) are employed for photothermal conversion. The organic materials based on cyanine, squarine, phthalocyanine, and diketopyrrolopyrrole are common PAs for PTT due to their photo-responsive properties and availability via chemical synthesis [92,93]. Metallic nanostructures, metal-oxide nanoparticles, carbon-based materials, and transition metal dichalcogenide nanostructures are examples of inorganic PAs [93,94]. It should be noted that metallic nanostructured PAs may quickly build up in the body or organs, leading to increased oxidative stress, inflammatory cytokine formation, and eventually cellular death [95]. MNPs have built their own following in PTT among a wide variety of inorganic materials used, as well as in combination cancer therapy as discussed in this review. Many forms of MNPs are capable of converting light to heat. For example, magnetic ternary nanostructures (mainly Cu–Co–S, Cu-Fe–Se, and Cu–Fe–S nanostructures) may be used as photothermal transducers [96]. MNPs such as iron oxide have increasingly shown their importance in NIR-laser-induced cancer PTT, due to their unique properties, such as their superparamagnetic nature, biocompatibility, ease of synthesis, availability for MR imaging and diagnosis, magnetic targeting, and good photo-absorption ability [97]. In addition, iron oxides have been approved by the US Food and Drug Administration (FDA) for clinical use, considering that iron is a quickly metabolized nutrient via cellular control through the transferrin pathway [94]. The magnetite (Fe_3_O_4_) is the most common form of iron oxide for this purpose, although with a propensity to be oxidized, thereby altering its magnetic properties [98,99]. As a result, biocompatible coatings such as polymers, silica, or gold (Au) are typically applied to iron oxide nanoparticles. The MNPs will be discussed for PTT below from two standpoints—fMNPs with MNPs alone and fMNPs modified with additional photothermal or plasmonic agents (hybrid MNPs). Numerous review articles have also addressed the importance of MNPs in PTT [11,30,100].

### 4.1. Functionalized Iron Oxide MNPs (fMNPs)

The use of NIR laser in thermal cancer therapies provides advantages over the use of AMF, as the latter requires a high current and voltage, in addition to its inability to focus on a specific region of interest within the applied magnetic field [101]. Furthermore, the biological window is open when using NIR lasers for PTT, considering the spectral range in which tissues become partially transparent due to the reductions in absorption and dispersion or scattering [30,102]. Human tissues experience high excitation near the visible region of the optical spectrum; thus, using a particular wavelength of light near the biological window for PTT will protect the healthy tissues or cells from adverse effects caused by unwanted light absorption [30]. The NIR light can penetrate biological tissues more effectively than visible light because tissues scatter and absorb less light at longer wavelengths. The light within the first biological window (NIR-I), with wavelengths ranging from 650 to 950 nm, is much superior to visible light for in vivo applications. Because longer wavelengths minimize tissue photon dispersion and background interference, light in the second biological window (NIR-II) operating in the 1000–1700 nm wavelength range has been shown to improve the detection sensitivity, spatial resolution, and tissue penetration depth [30,101,102]. Liao et al. reported a simple ligand-assisted hydrothermal reaction by building NIR-activated Fe_3_O_4_ nanostructures and tested them with an NIR laser (808 nm) in vitro with KB cells [103]. Chu et al. investigated in vitro and in vivo PPT approaches with Fe_3_O_4_ nanoparticles of various shapes using an 808 nm NIR laser light [104]. Co-precipitation and thermal decomposition methods are used to create spherical, hexagonal, and wire-like Fe_3_O_4_ MNPs with diameters of approximately 9.1, 9.4, and 12.6 nm, respectively. According to a study of the photothermal effects with NIR lasers, all three types of Fe_3_O_4_ MNPs can generate heat and increase the temperature by about 25 °C at an 0.8 mg/mL Fe_3_O_4_ dosage when irradiated with 808 nm laser light for 21 min. An in vitro study on esophageal cancer cells revealed that the viability was reduced to 52% using 0.5 mg/mL of Fe_3_O_4_ after being irradiated with NIR laser for 20 min. The NIR-induced PPT of spherical Fe_3_O_4_ MNPs, surface-modified with DSPE-PEG-COOH, can effectively prevent tumor growth in vivo using a human esophageal cancer model in xenograft mice [105]. Cabana et al. compared two types of Fe_3_O_4_ nanoparticles, both spherical and flower-like, with AMF-induced MHT and NIR-laser-induced PTT near the NIR-II biological window [101]. At lower concentrations, PTT tends to be more selective than MHT for both spherical and flower-like Fe_3_O_4_ nanoparticles. After 10 min of NIR laser (1064 nm) induction of spherical (or flower-like) magnetite nanoparticles at a concentration of 32 mM Fe, the temperature rose to 54 °C (or 56 °C) at 1 W/cm^2^ and 22 °C (or 24 °C) at 0.3 W/cm^2^. In comparison, MHT with spherical magnetite nanoparticles only increased the temperature by 6 °C after 10 min of AMF induction at 8 mT and 450 kHz, while flower-like particles showed higher temperature increases at 13 °C. Furthermore, the flower-like nanoparticles showed higher intracellular uptake than spherical nanoparticles. The antitumor thermal effect in cancer cells induced with NIR lasers at the lowest intensity (0.3 W/cm^2^) showed almost complete cell destruction with the same concentration of both Fe_3_O_4_ nanoparticles. Kharey et al. developed a single-step green synthesis method for producing eugenate (4-allyl-2-methoxyphenolate)-capped iron oxide MNPs from the medicinal aromatic plant Pimenta dioica. These nanoparticles showed significant heat generation after irradiation with a 1060 nm laser for PTT and were found to be safe for human cervical cancer (HeLa) and human embryonic kidney 293 (HEK 293) cell lines [106]. 

Several factors influence the heat output of MNPs from NIR light, including the light irradiation strength, as well as the concentration, shape, and size of MNPs. Clustered and flower-like MNPs can produce significantly more heat than monodispersed or spherical MNPs. Despite this, the safe limit for the light irradiation intensity in PTT using NIR-I or NIR-II is 0.3 W/cm^2^, which represents a major limitation in PTT [93,101]. Huang et al. used hydrazine-assisted reduction and a hydrothermal environment to create single Fe_3_O_4_ MNPs consisting of several aggregated Fe_3_O_4_ clustered nanoparticles, with saturation magnetization of up to 113 emu/g [Fe] and relaxivity of 234.6 mM^−1^ s ^−1^ [107]. After 10 min of irradiation with a 1064 nm laser (NIR-II) at a low power intensity level of 0.38 W/cm^2^ on clustered Fe_3_O_4_ nanoparticles (375 ppm [Fe]), the temperature rose to 58 °C from 25 °C. They also visualized clustered MNP-treated cells using optical coherence tomography after exposure to a 860 nm laser (NIR-I). The study demonstrated that MNPs could be moved with magnetic force, suggesting they are a promising optical contrast agent for dynamic medical imaging. Significant cancer cell death was observed after incubating the HeLa cells with clustered Fe_3_O_4_ MNPs, which then irradiated with an 1064 nm laser. Shen et al. investigated the photothermal effect of clustered versus individual Fe_3_O_4_ MNPs in another study [95]. According to both the in vitro and in vivo tests, clustered Fe_3_O_4_ nanoparticles have a better photo-absorbing effect in the NIR-I window and a better therapeutic effect than individual ones. From the in vitro photothermal response studies, the temperature of clustered Fe_3_O_4_ nanoparticles (80 µg/mL) managed to reach 55 °C from 25 °C after 3 min of irradiation with an 808 nm NIR laser (5 W/cm^2^), while individual Fe_3_O_4_ nanoparticles only managed to reach 50 °C. For the in vivo photothermal effects, 25 µL of clustered or individual Fe_3_O_4_ nanoparticles (2 mg/mL) was administered by IT injection into tumor-bearing mice and then irradiated with an NIR laser at 5 W/cm^2^. After 2 min for the clustered Fe_3_O_4_ group and 3 min for the individual Fe_3_O_4_ group, the temperatures increased to 55 °C and 50 °C, respectively. This study again endorsed the improved photothermal therapeutic performance using clustered Fe_3_O_4_ nanoparticles. 

**Figure 3:**
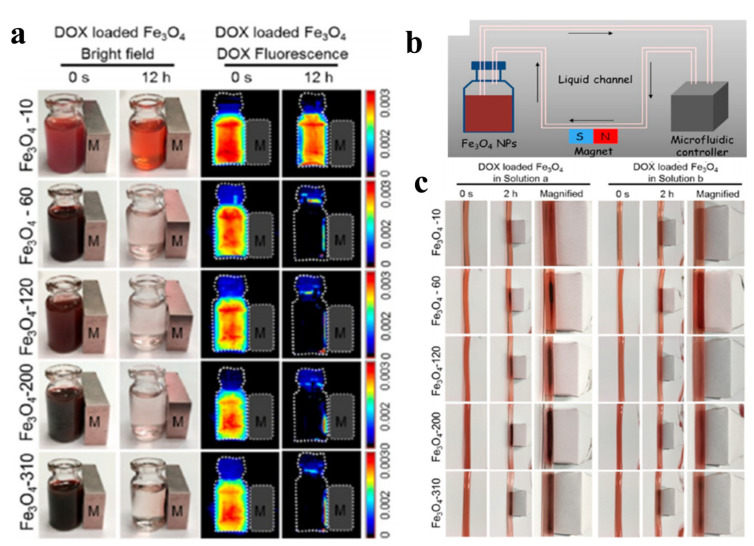
The in vitro magnetic responsiveness of iron oxide magnetic nanoparticles (MNPs) measuring 10 nm (Fe_3_O_4_-10), 60 nm (Fe_3_O_4_-60), 120 nm (Fe_3_O_4_-120), 200 nm (Fe_3_O_4_-200), and 310 nm (Fe_3_O_4_-310). (**a**) The images of fluorescent DOX-loaded MNPs before and after magnetic targeting with an external magnetic field in static mode. (**b**) A schematic diagram showing magnetic targeting in dynamic mode with a microfluidic system simulating nanoparticle retention in blood circulation, under the guidance of an external magnetic field. (**c**) The time-lapsed photographs of the magnetic retention of different nanoparticles in the flow system shown in (**b**) at the 32.85 cm/s flow rate found in the artery. Reprinted/adapted with permission from Ref. [97]. 2017, American Chemical Society.

The PTT-based thermal ablation of tumor cells is a reliable local treatment technique. However, although non-targeted tissues can be minimized with the local administration of MNPs and NIR laser-induced PTT, it is difficult to completely eliminate large tumors with traditional PTT due to the residual tumor mass involved in the treatment regime [108]. Furthermore, the heat generated by an external stimulus can cause blood coagulation in the tumor vessels. When additional chemotherapy is combined with PTT, the anticancer drug will function intracellularly, resulting in synergistic therapeutic effects for cancer treatment [109]. While these combination treatment techniques have been widely investigated to improve their overall effectiveness, the leading site of their intervention is limited to local tumors, and the use of PTT against metastatic tumors disseminated from the source of NIR remains impracticable [108]. Nonetheless, some research has shown that combining PTT with chemotherapy not only effectively prevents the primary tumor growth, but also dampen the metastasis [108,110]. Iron oxides can influence the tumor microenvironment through a biological effect mediated by nanoparticles. It has been discovered that they polarize the pro-inflammatory M1 macrophages. In wounds, pro-inflammatory M1 macrophages emit hydrogen peroxides, which elicit iron and initiate the Fenton reaction, producing highly toxic hydroxyl radicals (•OH). Furthermore, iron oxide has also been shown to convert M2-subtype tumor-associated macrophages (TAM) to M1-subtype TAM and to reroute tumor-associated immunocytes, thereby inhibiting tumor development. Li et al. used bare iron oxide as a photothermal agent, co-loaded with an anticancer drug HCPT (10-hydroxy camptothecin) within a nanogel for combination chemo-PTT [110]. The hybrid nanogel demonstrated a synergistic antitumor effect in a MCF-7 breast cancer cell-bearing mouse model, which was further improved by the additional targeting effect offered by an external magnetic field. To investigate the chemo-photothermal effects on metastasis inhibition mediated by the hybrid nanogel, a high-metastasis breast cancer model was developed using 4T1 cancer cells. Metastasis nodes in the lung tissues shown by H&E staining indicated that the combination of chemotherapy with PTT, by inducing the hybrid nanogel with NIR light, can prevent primary tumor growth and mitigate metastasis. As previously stated, MNPs are responsive to an external magnetic field for specific tumor-targeted delivery, and the efficiency of their external magnetic-field-guided targeting is dependent on their physicochemical properties, particularly the particle size. Toward this end, Guo et al. evaluated the magnetic targeting ability of iron oxide MNPs in various sizes ranging from 10 to 310 nm. The MNPs were functionalized by covalent binding with carboxymethyl chitosan for loading of the chemodrug DOX through electrostatic interactions. The in vitro magnetic responsiveness was studied in static mode and in dynamic mode with a microfluidic system simulating nanoparticle retention in blood circulation under the guidance of an external magnetic field (Figure 3) [97]. They also conducted in vitro magnetic targeting studies using MCF-7 cells and an in vivo antitumor study using subcutaneously implanted S180 cells in nude mice. It was demonstrated that larger Fe_3_O_4_ fMNPs exhibited better magnetic-targeting efficiency when guided by an external magnetic field. The larger MNPs also demonstrated a more potent in vitro or in vivo magnetic response, with increased nanoparticle accumulation in the tumor. The strongest antitumor efficacy was shown by using 310 nm MNPs for a combination PTT–chemotherapy. 

Due to the partially filled d-orbitals and changing oxidation states, iron oxide plays a prominent redox–catalysis function in many energy transfer processes, being intimately related to the reactive oxygen species’ (ROS) chemistry and consequently affecting the biological tissue or cells through Fenton reactions [111]. One study involved coating two forms of iron oxide MNPs with polydopamine and PAMAM dendrimers and functionalizing them with N-hydroxysuccinimide–polyethylene glycol–maleimide (NHS-PEG-Mal) and folic acid for the in vitro chemo-PTT of hepatocellular carcinoma [112]. Both regular and spherical iron oxide MNPs demonstrated excellent photothermal properties and ROS production, which aid in oxidative stress and increase the cytotoxicity. However, the spherical MNPs showed an improved photothermal response and efficient antitumor effect. In another study, Zhu et al. investigated the use of enzyme-responsive MNPs in chemo-PTT–photodynamic therapy (PDT) [113]. As a photothermal agent, iron oxide MNPs were modified with CuS, which can produce cytotoxic ROS. In addition, gelatin was conjugated to the MNPs for gelatinase-responsive DOX release, as gelatinase is a common endogenous proteolytic enzyme in tumor tissues for the digestion of gelatin. After NIR laser exposure, the MNPs showed a synergistic chemo-PTT-PDT effect when compared to individual therapies. In another study, Dorjsuren et al. investigated a thermosensitive liposomal system co-entrapping MNPs and DOX and surface-modified with an EGFR antibody (CET) (CET-TSMDLs) in breast cancer therapy [114]. From both in vitro and in vivo studies, the developed CET-TSMDLs could slow down the tumor growth for combination chemo-PTT. 

### 4.2. Hybrid-Material-Functionalized MNPs

Over the last decade, gold nanoparticles (AuNPs) have been the choice as light-responsive nanomaterials for PTT. AuNPs can turn optical energy into heat, which causes the surrounding medium’s temperature to rise. AuNPs with spherical shapes respond to a shorter wavelength range (~520 nm), i.e., below biological window I, than the NIR region (biological window I/II). The AuNPs can be designed as nanorods, nanoshells, nanocages, nanopopcorn, or hollow nanospheres, which can improve the tissue penetration depth for PTT [115,116]. As AuNPs do not photo bleach, an Au coating layer may stabilize the MNPs and prevent environmental erosion, while the Au-modified layer will show lower cellular toxicity towards normal cells [116]. Several studies have investigated MNP-capped Au (core–shell) nanoparticles, in which the surface coating of MNPs with Au can increase the temperature synergistically upon NIR laser irradiation. For example, Abed et al. studied magnetically targeted PTT with core–shell iron oxide–Au nanoparticles [115]. These multifunctional theranostic platforms have been studied for their antitumor efficiency in a CT26 colorectal tumor-bearing mice model. With the IV administration of core–shell hybrid nanoparticles followed by targeting with a permanent magnet at the tumor site and NIR laser irradiation, the tumor growth was suppressed. Pandesh et al. conducted a similar study in B16-F10 tumor-bearing mice with magnetically targeted delivery [117]. In another study, core–shell iron oxide–Au nanoparticles were investigated as PS and MRI contrast agents. The in vitro experiments revealed that hybrid MNPs have negligible cytotoxicity in KB cells, while after NIR laser exposure approximately 70% of cells are dead [118]. Guo et al. showed that hybrid MNP–Au core–shell nanoparticles could increase phagocytosis in pancreas cancer cells, thereby improving the MRI contrast. With increasing concentrations of core–shell nanoparticles or applied NIR laser power intensities, the growth of pancreatic cancer cells was drastically reduced [119]. 

To be useful in theranostics, where both diagnostic and treatment modalities are integrated into a single platform, the PA for PTT must be able to assist in diagnostic imaging, in addition to having the basic function of destroying cancer cells via their photothermal effects. Like AuNPs, MNP-capped Au core–shell nanoparticles are magnetic–plasmonic nanoparticles (MPNP) and are useful as contrast agents in bio-imaging. For improved stability, such core–shell MPNPs can be further modified with bio-compactable polymers. This can reduce the aggregation, improve the dispersion and colloidal stability, and prevent unfavorable surface oxidation. Furthermore, the use of a charged polymer coating on the surface of the MPNPs improves their steric stabilization via repulsive forces [120]. For instance, polymer nanocomposites, such as polypyrrole (PPY), have emerged as promising candidates in the field of drug delivery due to their high conductivity, low toxicity, and good biocompatibility. An improved theranostic agent can be designed using the inherent properties of SPIONs, Au and PPY. Feng et al. investigated these combinations for cancer treatment and discovered that Au/PPY/Fe_3_O_4_ demonstrated good colloidal stability, strong NIR absorbance, bio-stability, and low cytotoxicity. Their research showed that the highly versatile multifunctional Au/PPY/Fe_3_O_4_ nanocomposites show potential as theranostics agent for simultaneous cancer diagnostic imaging (MRI and X-ray computed tomography) and cancer therapy [121]. In another study, Bhana et al. used the seed-mediated growth method to create a self-assembled Fe_3_O_4_ cluster core with Au shells. A polymer coating with silicon 2,3-naphthalocyanine-dihydroxide and stabilization with 11-mercaptoundecanoic-linked PEG were applied to this nanocomposite. The polymer-coated core–shell nanopopcorn demonstrated greater efficacy while avoiding systemic toxicity, indicating that dual-mode PTT and PDT treatment with the assistance of magnetic-field-guided targeted delivery significantly improves the therapeutic efficacy when compared to the combination treatment without the use of a magnetic field [116]. Abedin et al. demonstrated that surface-modifying the iron oxide core and Au shell nanostructure with the polymer poly-l-lysine (PLL) can improve its internalization by mammalian cells electrostatically due to the presence of the PLL (a positively charged polymer), as well as the colloidal stability of the dispersion in aqueous solutions such as H_2_O, PBS, PBS + 10% fetal bovine serum (FBS), and cell culture medium [120]. Along this line, Riva et al. also demonstrated that the surface modification of the MPNPs with polymers or silica does not influence their physical or thermal properties but rather increases their stability [122]. To this end, a positively charged polymer polyethylenimine (PEI)-functionalized silica embedded with MNPs was electrostatically modified with Au nanorods, before being modified with bovine serum albumin (BSA). In another study, Ohulchanskyy et al. created magnetic–plasmonic phospholipid micelles (MPPM) by entrapping Au nanorods and MNPs to investigate magnetic-field- and image-guided delivery in NIR-laser-induced PTT [123]. As expected, the results showed that greater colloidal stability in a biological medium as well as increased accumulation of MPPM in the cells with magnetic targeting can be achieved. The Au nanorods can respond to NIR light in femtoseconds to generate cell-destructive nanobubbles inside cells. As a result, the combination of Au nanorods and MNPs in a single magnetic-driven nanoplatform improves the PTT. In another study, Bertorelle et al. conducted in vitro studies with D2B-antibody-modified MPNPs for targeted photothermal treatment with human prostate cells [124]. D2B is a novel monoclonal antibody that specifically targets the extracellular domain of the prostate-specific membrane antigen (PSMA), which is overexpressed in prostate cancer. Additionally, because the absorbance of the Au nanorods is near the NIR region, photoacoustic imaging (PAI) is possible [123]. Several studies have also been conducted to use MPNPs for enhanced imaging and photo-induced therapy. Mehrmohammadi et al. studied pulsed magneto-motive ultrasound (PMMU) imaging and demonstrated signal amplification from clustered MNPs vs. individual MNPs [125]. They also reported the detection of the intracellular accumulation of MNPs in macrophages using PMMU [126]. 

Combination cancer therapies, as opposed to individual treatments such as PTT or chemotherapy, can improve the antitumor effects. Li et al., for example, investigated a chemo-photothermal co-therapy with multifunctional nanocomposite materials for cancer diagnosis, targeting, and treatment [127]. The MS-doped Mn^2+^ and DOX-loaded nanoparticles were further modified with HA ligand and conjugated with Au nanorods to synthesize MSMnD/HA-GNR. The Mn^2+^ and Au nanorods act as MRI and CT contrast agents, respectively, in addition to as a PA, while HA acts as a targeting agent, and DOX as a chemotherapeutic agent. The in vitro results revealed that the MSMnD/HA-GNR nanocomposite has excellent colloidal stability and biocompatibility, as well as outstanding ablation ability for murine lymphatic tumors when combined with laser irradiation. In another study, Peng et al. created DOX-entrapped MS magnetic Au nanoclusters (DMSMGNCs) for magnetic targeted delivery and diagnosis, as well as for chemo-PTT co-therapy in 4T1 cancer-bearing Balb/C mice [109]. DMSMGNCs showed a good photothermal response and burst drug release upon irradiation with an NIR laser and exhibited external magnetic field-guided particle accumulation at the tumor site. Further, DMSMGNCs combined with magnetic targeting for chemo-PTT co-therapy resulted in effective tumor growth suppression and mediastinal metastasis prevention. The MNP-Au core–shell nanoparticles may also combine with other PAs. Using ICG as a PS, Kwon et al. investigated the anticancer effects of temozolomide (TMZ) and ICG-loaded Fe_3_O_4_ nanoparticles (TMZ-ICG-Fe_3_O_4_) for chemo-PTT with U87MG human glioblastoma cells [128]. Under NIR laser irradiation, the ICG-embedded Fe_3_O_4_ MNPs demonstrated an excellent photothermal effect and photo-stability. Furthermore, when used in chemo-PTT, TMZ- and ICG-loaded Fe_3_O_4_ nanoparticles demonstrated synergistic cell cytotoxicity. The results from the experiments demonstrated that the combination chemo-PTT with TMZ-ICG-Fe_3_O_4_ nanoparticles induced effective cancer cell death mediated by increased ROS generation, and modulated both the intrinsic and extrinsic apoptotic pathways (Bcl-2, cytochrome C, CP-3, and CP-8), as revealed by quantitative reverse transcription–polymerase chain reaction (qRT-PCR) and Western blot studies. In another study, a dual-target nanomedicine consisting of pH-sensitive SPIONs and Au core–shell nanoparticles modified with chitosan and folate was developed for the delivery of DOX. This versatile nanoparticle made of biocompatible hybrid materials (SPIONs, Au, CS, and FA) could lower DOX’s toxicity for targeted cancer therapy [129]. A summary of the studies using functionalized MNPs for NIR-laser-induced PTT and chemo-PTT is provided in Table 2.

## 5. NIR- and AMF-Induced Magneto-Photo-Hyperthermia (MPHT)

**Figure 4:**
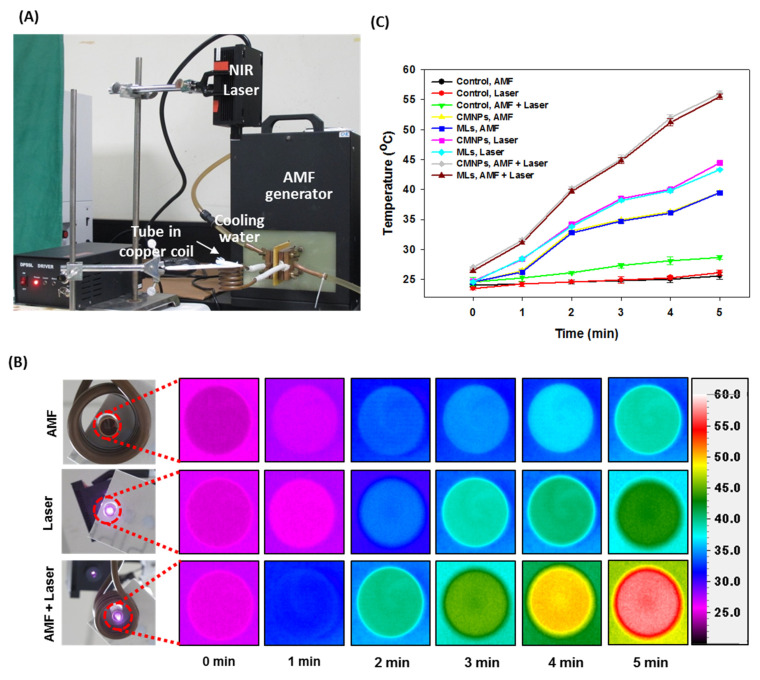
The in vitro heating efficiency of CMNPs and MLs as induced by magnetic hyperthermia–photothermia [130]. A 500 μL solution of CMNPs or MLs prepared in distilled water (corresponding to 0.6 mg/mL CMNPs) was taken in a 2 mL Eppendorf tube and subjected to AMF or NIR laser treatment. The tube was placed in the center of a 30.5 mm internal diameter solenoid copper coil for AMF induction at 52 kHz or 808 nm NIR laser exposure at 1.8 W/cm^2^ from the top (**A**). The time-lapsed thermal images were acquired from the tube bottom with an infrared thermal camera (**B**) and the peak temperature was plotted as a function of the treatment time (**C**). The control was distilled water without CMNPs.

As previously stated, cancer therapy where MNPs are induced with an AMF is known as MHT; therapy with MNPs induced by NIR lasers is known as PTT, and therapy with MNPs induced with both an AMF and NIR lasers is known as magneto-photo-hyperthermia (MPHT). However, only a few studies have been conducted for MPHT. The in vitro studies on MPHT discovered that combining AMF and NIR laser treatments increased the thermal effect synergistically (Figure 4) [130]. In the study, citric-acid-coated iron oxide magnetic nanoparticles (CMNPs) were loaded into cationic liposomes to form magnetic liposomes (MLs), and the encapsulation efficiency of the CMNPs and size of the MLs were optimized using the response surface methodology. For thermal effect studies, the lowest concentration (0.6 mg/mL) of MLs was chosen and induced for 5 min with an AMF (52 kHz) and NIR lasers (1.8 W/cm^2^), either individually or concurrently. Regardless of the treatment mode, there were no statistically significant differences in temperature between the CMNPs and MCLs; however, the AMF increased the temperature to 39 °C, the NIR lasers to 43 °C, and the AMF + NIR laser treatment to 56 °C from 25 °C. The higher temperature increase in the combination mode was due to the CMNPs being excited by absorbing light near the NIR-I region while also undergoing an AMF-induced dipole interaction. The cell cytotoxicity analysis with U87MG cells revealed that the combined MHT + PTT treatment had a synergistic effect on cancer cell death. In another paper, Espinosa et al. conducted in vitro and in vivo studies for dual-mode MPHT with cubic-shaped iron oxide nanoparticles (CINPs) [12]. The in vitro thermal studies revealed a synergistically improved thermal effect with a 15 °C temperature increase (ΔT) after inducing CINPs (25 mM [Fe]) with AMF (520 kHz, 5 min) at 9 °C after irradiation with NIR lasers (0.3 W/cm^2^, 5 min) at 25 °C in dual-mode (AMF + NIR-laser). The in vitro cell cytotoxicity experiments with SKOV3 cells for the dual-mode treatment revealed lower cell viability than in cells treated individually. In in vivo tumor thermal studies, nude mice were subcutaneously injected with A431 human epidermoid carcinoma cells, and MHT, PTT, and MPHT treatments were performed by injecting 50 µL of CINPs (4 mg/mL) intratumorally. Individual treatment with AMF (110 kHz) or NIR lasers (0.3 W/cm^2^) for 5 min resulted in the slight inhibition of tumor growth; nonetheless, dual-mode therapy results in complete tumor regression. 

Undoubtedly, the dual use of AMF and NIR lasers can solve the functional limitation related to the low heating efficiency of iron oxide MNPs, which could be further complemented with chemotherapy. Sharif et al. investigated dual-mode MPHT treatments for chemo-thermal combination studies, i.e., chemo-MHT, chemo-PTT, and chemo-MPHT [131]. Polyacrylic-acid-coated iron oxide MNPs with porous structures were used to load DOX and then modified with the targeting agent lactoferrin (Lf-DOX-PMNPs). Lactoferrin, which also has anticancer activity, causes apoptosis by downregulating Bcl-2 mRNA23 and upregulating Bax and caspase (CP)-3 mRNA. They found that the Lf-DOX-PMNPs can prolong the blood circulation of the drug after targeted drug delivery and can minimize drug resistance in cancer tissue. In vivo studies with mice bearing breast tumors (4T1) revealed that Lf-DOX-PMNPs mediated the chemo-MPHT effect by stimulating apoptosis in breast cancer cells through CP-3 activation, potentially decreasing the tumor growth by prolonging the drug availability to tumor cells. In another study, Lu et al. investigated a dual-mode antiglioma MPHT treatment by modifying MPNPs with CET (CET-MPNPs) [132]. The inhibitory and apoptotic rates of CET-MPNPs in U251 glioma cells in vitro were significantly higher in the combined MPHT group than in the individual MHT or PTT groups. The CP-3, CP-8, and CP-9 expression levels were significantly upregulated, demonstrating the effective antitumor effect by inducing intrinsic apoptosis. Consequently, compared to individual thermal treatment by MHT or PTT, the combined MPHT treatment mediated by CET-MPNPs inhibited the tumor growth significantly in vivo. The studies using functionalized MNPs for AMF- or NIR-laser-induced MPHT and chemo-MPHT are summarized in Table 3.

## 6. Conclusions and Outlook

Multifunctional nanomaterials integrating both cancer diagnosis and therapy functions into a single nanoplatform can provide a theranostic nanoagent for effective cancer treatment. The MNPs are excellent choices for thermal cancer therapies due to their versatile properties for theranostic purposes, as an MRI contrast agent for magnetic targeting, and as a photothermal agent triggered by an AMF or NIR lasers. The functionalization of MNPs with different moieties can improve their biocompatibility and broaden the treatment protocols in combination cancer therapy. However, changes in the size and shape of the nanocomposites must be taken into consideration for intracellular uptake. The behaviors of these nanocomposites may change during in vitro and in vivo studies and must be followed using comprehensive and detailed characterization techniques. The fMNPs with spherical and flower-like shapes were shown to have higher tumor uptake rates, while the clustered form can increase temperatures more than individual ones, with the latter showing higher colloidal stability. The magnetic targeting efficiency also varies according to the size of the MNPs, but larger MNPs usually respond faster to an external magnetic field than smaller ones, albeit with hindered intracellular uptake. Compared to individual magnetic hyperthermia or photothermal treatment, the combination treatment modalities are more promising, resulting in better synergistic treatment outcomes. The clinical limitations of AMF-induced MHT (magnetic field strength) or NIR-laser-induced PTT (light intensity) can be overcome with a dual-mode MPHT treatment combining an AMF and NIR laser. Nonetheless, a clinically feasible design for MPHT with fMNPs must be developed for translational nanomedicine research in cancer therapy.

## Figures and Tables

**Table 1:** Summary of the use of functionalized MNPs for AMF-induced cancer therapy.

Types and Size of MNPs	Functionalizing Agents	Cancer Cells	Types of Study	AMF	Reference
Maghemite (γ-Fe_2_O_3_), ~5 nm	MS	A549, HeLa, Saos-2, HepG2	In vitro	100 kHz, 15,916 A m^−1^	[47]
Iron oxide (Fe_3_O_4_), 10–20 nm	Silica	L929, HeLa	In vitro	250 kHz, 26,659 A m^−1^	[48]
Manganese ferrite, ~50 nm	Silica-RITC, silica	HeLa	In vitro	33.3 A m^−1^	[49]
SPIONs, ~15 nm	Silica microbeads	Co112	In vitro, in vivo	141 kHz	[50]
Iron oxide magnetic nanorings, ~70 nm	Polyethylene glycol methyl ether	MCF-7	In vitro, in vivo	39,789 A m^−1^ 400 kHz	[56]
Iron oxide magnetic nanorings ~70 nm	PD-L1, PEG	4T1	In vitro, in vivo	365 kHz, 30 kA m^−1^	[57]
Fe_3_O_4_ nanocube, ~30 nm	Chitosan oligosaccharide	A549	In vitro, in vivo	1 MHz, 208 A m^−1^	[58]
Iron oxide	PLGA	MDA-MB-231	In vitro, in vivo	513 kHz, 8 kW	[61]
Iron oxide	Polymethyl-methacrylate	MB-231	In vitro, in vivo	626 kHz, 28.6 A	[62]
Hexagonal cobalt and Manganese-doped MNPs, ~20 nm	Poly-ethylene glycol)-b-poly-caprolactone (PEG-PCL), SiNc	ES-2	In vitro, in vivo	420 kHz, 27 kA m^−1^	[63]
Mn–Zn ferrite magnetic nanocryastals, ~14 nm	Phospholipid-PEG, ICG, RGD	4T1	In vitro, in vivo	390 kHz, 2.6 kA m^−1^	[76]
Zn-doped iron oxide, ~15 nm	Au, ATAP	U87, MCF-7	In vitro	300 kHz, 5 kA m^−1^,	[77]
Ferric-oxide MNPs, ~70 nm	HER2 aptamers, dextran	SK-BR3, U87MG	In vitro	280 kHz, 300 A	[78]
Iron oxide	Calcium phosphate cements	MB-231	In vitro, in vivo	626 kHz, 28.6 A	[86]
Fe_3_O_4_ nanocube, ~20 nm	Serum albumin	U87MG	In vitro, in vivo	512 kHz, 10 kA m^−1^	[87]
Magnetite MNPs, ~8, 17, and 24 nm	Human-like collagen protein (HCP)	BHK-21	In vitro, in vivo	360 kHz, 44,564 A m^−1^	[88]
SPIONs, ~15 nm	Carboxyl-modified DNA20, MS, DOX	HeLa	In vitro	141 kHz	[51]
Magnetic MS, ~190 nm	NIPAM-co-MAA, MS, DOX	HeLa	In vitro	18 mT, 409 kHz	[52]
Maghemite, 16 nm	PEI/NIPAM, MS, soybean trypsin inhibitor	-	Drug release	24 kA m^−1^, 100 kHz	[53]
Iron oxide crystals, ~190 nm	Metal–organic framework-ZIF-90, PDA, DOX	HeLa	In vitro	18 mT, 490 kHz	[67]
Maghemite and magnetite magnetic nanorods, ~64 to 530 nm	Poly(ethyleneimine), Poly(sodium 4-styrenesulfonate), DOX	-	Drug release	10–20 kA m^−1^, 100–200 kHz	[68]
Mn-Zn ferrite MNPs, ~100 nm	PLA-b-poly(N-co-D), CPT	SK-OV-3, HepG2	In vitro	89.9 kA m^−1^, 114 kHz	[72]
Mn-Zn ferrite MNPs, ~100 nm	6sPCL-b-P(MEO2MA-co-OEGMA), DOX	Huh-7	In vitro, in vivo	89.9 kA m^−1^, 114 kHz	[73]
Citric-acid capped iron-oxide, ~12 nm	Liposomes, CET, CPT	U87MG	In vitro, in vivo	96 kHz, 60 A	[85]
Iron oxide nanocubes, 15 nm and 23 nm	Polycaprolactone nanofibers, DOX	HeLa, MCF-7	In vitro	110 kHz, 30 kA m^−1^	[91]

**Table 2:** Summaries of studies using functionalized MNPs for NIR-induced cancer therapy.

Types of MNPs	Functionalizing Agents	Cancer Cells	Type of Study	NIR Light		Reference
				Wavelength (nm)	Intensity (W cm^−2^)	
Spherical, hexagonal, and wire-like Fe_3_O_4_	DSPE-PEG-COOH	Eca-10	In vitro, in vivo	655, 671, 808	-	[104]
Iron oxide	Carboxymethyl chitosan, DOX	MCF-7	In vitro, in vivo	808	1.5	[97]
Regular and sphere shape Fe_3_O_4_	NHS-PEG-Mal, folic-acid, DOX	HepG2	In vitro	808	2	[112]
Iron oxide	CuS, DOX	MCF-7	In vitro, in vivo	980	2	[113]
Citric-acid-capped iron-oxide	CET, TSLs, DOX	SKBR-3, MCF-7	In vitro, in vivo	808	2	[114]
Fe_3_O_4_-core Au shell	PPY	HeLa	In vitro	808	2	[121]
Individual and clustered Fe_3_O_4_	-	A549	In vitro, in vivo	808	5	[95]
CuFeSe2 nanocrystals	poly(methacrylic acid)	4T1	In vitro, in vivo	808	1	[96]
Magnetite/maghemite nanospheres and nanoflowers	-	SKOV-3, PC3	In vitro	1064	0.3, 1	[101]
Oleate-Fe_3_O_4_ and pristine Fe_3_O_4_	Peptide	4T1	In vitro, in vivo	808	2	[105]
Clustered Fe_3_O_4_	Calcium oxalate dehydrate	HeLa	In vitro	808, 1064	0.38	[107]
Oleic-acid capped Fe_3_O_4_	10-Hydroxy camptothecin, NIPAm, MAA, mPEGMA	MCF-7, 4T1	In vitro, in vivo	808	3	[110]
Fe_3_O_4_	Core–shell (Fe_2_O_3_@Au)	CT26	In vitro, in vivo	808	1.4	[115]
Clustered Fe_3_O_4_	Au nanopopcorns, PEG	KB-3-1, SK-BR-3	In vitro	808	0.55	[116]
Fe_3_O_3_@Au (core–shell)	-	B16-F10	In vivo	808	2.5	[117]
Fe_3_O_4_@Au (core–shell)	-	KB	In vitro	808	6.3	[118]
Fe_3_O_4_@Au (core–shell)	Poly-L-lysine	BT-474, MDA-MB-231	In vitro	808	1	[120]
Fe_3_O_4_@Au nanords	HA, MS, DOX	HCT 116, HEK 293	In vitro, in vivo	980	3	[127]
Fe_3_O_4_@SiO_2_ (core–shell)	PEI-FPBA, ICG, TMZ	U87 MG	In vitro	808	1	[128]

**Table 3:** Summary of the use of functionalized MNPs for AMF- or NIR-laser-induced cancer therapy.

Types of MNPs	Functionalizing Agents	Cancer Cells	Type of Study	AMF	NIR Light		Reference
					Wavelength (nm)	Intensity (W cm^−2^)	
Fe_3_O_4_ nanocubes	-	SKOV3, PC3, A431	In vitro, in vivo	110 kHz	808	0.3	[12]
Citric-acid-capped Fe_3_O_4_	Cationic liposome	U87MG	In vitro	52 kHz	808	1.8	[130]
Fe_3_O_4_	Poly-acrylic acid, lactoferrin, DOX	4T1	In vitro, in vivo	540 kHz	808	5	[131]
Fe_3_O_4_@Au (core–shell)	CET	U251	In vitro, in vivo	230 kHz	635	0.3	[132]
